# Saudi Arabian Perceptions of Childhood Anxiety, Parental Communication, and Parenting Style

**DOI:** 10.3390/healthcare11081142

**Published:** 2023-04-16

**Authors:** Lowai G. Abed, Mohaned G. Abed, Todd K. Shackelford

**Affiliations:** 1 Department of Communication and Public Relations, College of Communication and Media, University of Jeddah, Jeddah 21589, Saudi Arabia; lgabed@uj.edu.sa; 2 Department of Special Education, Faculty of Education, King Abdulaziz University, Jeddah 21589, Saudi Arabia; mabed@kau.edu.sa; 3 Department of Psychology, Oakland University, Rochester, MI 48309, USA

**Keywords:** childhood anxiety, communication, parenting style, authoritative behavior, authoritarian behavior, learning, modeling, Saudi Arabia

## Abstract

Parenting communication and parenting style, including expressions of fear, worry, and threat, may contribute to children’s anxiety and apprehension. This study examined the degree to which perceptions of parental communication (both verbal and nonverbal) and parenting style are linked with childhood anxiety. This is among the first studies to investigate these relationships in a Saudi Arabian context. We employed a sample of 121 Saudi adults who completed questionnaires measuring perceptions of 2 parenting styles (authoritative and authoritarian), parental anxiety, and childhood anxiety. Parental communication elements such as shouting, criticism, facial expression, and body expressions were included among assessments of perceived parental anxiety, authoritative style, and authoritarian style. The results indicated that perceptions of childhood anxiety were positively associated with parental anxiety but not consistently associated with other assessed variables. This research addressed perceptions of parental communication and parenting style in the development of childhood anxiety, extending upon previous research on Western samples to a Middle Eastern sample residing in Saudi Arabia.

## 1. Introduction

Parents guide their children with instructions that are shared through communication, both verbal and nonverbal [1]. As parents communicate with their children, they express their feelings, emotions, attitudes, behaviors, and personalities. Therefore, parents’ communication is influenced by their dispositions, and this, in turn, has the capacity to affect their children [1,2]. The current study focused on anxiety, which is one of the conditions or traits that can be displayed and expressed while communicating. In linking anxiety to parental communication, this research focused on parental behavior and personalities. How parents communicate with their children expresses their personalities, especially if conducted consistently. This raises the question as to whether anxiety in children is a normal occurrence and/or is influenced by those close to them, such as parents [2,3,4]. This subject highlights the significance of communication not only in instructing but also in affecting conditions and behaviors in others, especially among close relations.

Of particular interest are two parenting styles that affect how a parent communicates, namely the authoritative style and the authoritarian style [4,5]. An authoritative parenting style is characterized by low responsiveness and high demands, and parents who apply this style are often hostile and employ punishments to gain compliance [5,6]. An authoritarian parenting style is characterized by firm control to the extent that parents do not force their opinions on their child, enforce minimal restrictions, or acknowledge the child’s interests and feelings [5,7]. Parents displaying either of these styles might affect their child’s anxiety regardless of whether the parent suffers from anxiety. Here, the focal point is how communication occurs between the child and parent based on parenting style. Some of the factors to consider are whether parents are intrusive, controlling, and regulating of the child’s behavior; employ strict disciplinary strategies and harsh behavioral controls; or are involved, accepting, and responsive toward the child, offering support. One of the assumptions of the current study is that authoritative and authoritarian parenting styles are consistent enough to affect childhood anxiety. Parenting style can also be expressed through varying levels of psychological control. According to Kuppens and Ceulemans [8], parental psychological control is intrusive and sometimes includes attempts to manipulate a child’s thoughts, emotions, and feelings. As such, manipulative tendencies can lead to psychological control that is linked with negative developmental outcomes such as depression, antisocial behaviors, and anxiety in children [9,10]. Thus, parenting style and the manner in which it is expressed or communicated may be associated with psychological effects and outcomes in children.

### 1.1. Literature Review

Studies that have explored the role of parental communication in contributing to childhood anxiety have focused on verbal or nonverbal communication. These two forms of communication are both important in instructing and creating perceptions in children, although most studies have focused on parental verbal communication. Percy et al. [11] found that a parent’s verbal communication containing expressions of fear-relevant information (such as a warning about a dangerous location) may produce anxiety in children. The same study also found that genetic differences account for about one-third of the risk-related variance of anxiety, with the remainder accounted for by external influences [11]. Fear-relevant verbal communication entails messages that unequivocally designate threat. For instance, messages that unreservedly indicate threat through emphasis on susceptibility, such as “…you’ll be terrified”, or messages that suggest avoiding an alarming situation, such as “…don’t reach the edge of the river”, can verbally transmit anxiety. According to Percy et al. [11], verbal communication can sometimes have an over-controlling function that can limit a child’s opportunities to achieve mastery over external factors. The significance of verbal communication in transmitting fear and anxiety has gained support from research with children. For instance, Hoffman et al. [12] established that children who experience fear-related verbal communication in controlled conditions develop anxious behavior, whereas positive verbal communication has a contrary effect. Similarly, Leigh and Clark [13] found that children develop fear and anxiety when consistently and repeatedly exposed to fear- or threat-related information.

Recent research suggests possible environmental conduits for the parent-to-child transmission of generalized anxiety. Aktar et al. [14] presented evidence that children acquire generalized anxiety and avoidant reactions to possible threats from parents through information and modeling. This is because parents with generalized anxiety may prejudice their children’s processing of possible threats in the external setting by transmitting messages that the world is unsafe, that children should avoid strong emotions, and that worrying about situations helps individuals manage uncertainty [14]. In such cases, the interactions between danger and resilience factors may shape adjustment in children. Therefore, the external influences linked to parental anxiety are among the risk factors that can affect childhood anxiety, in addition to other danger and resilience-promoting facets of the child and the external setting. Burstein and Ginsburg [15] conducted a study of parental modeling and anxious behavior in children and found that children reported higher anxiety and preferred avoidance habits in anxious settings relative to nonanxious settings. The study focused on identifying the external features that serve as risks for childhood anxiety that could provide prospective guidance for intervention programs. For instance, the recognition of external factors affecting child development such as parenting style might direct the enhancement in existing interventions as well as facilitate the development of new interventions to treat childhood anxiety.

Most studies have investigated the role of parenting style as a potential influence on anxiety development in children, with fewer studies focusing on parental modeling. Some of the focus points of the former include over-controlling behavior expressed by parents (i.e., authoritarian style), parental positivity, and parental responsiveness (i.e., authoritative style). Aktar et al. [14] determined that parental modeling of anxious habits such as avoidance and parental expression such as communicating potential threats or distrust in a child’s ability to handle situations may be important for understanding the development of anxiety in children. Unfortunately, parental modeling has not been a major focus of past studies despite its potential influence on anxiety development. Zhang et al. [16] investigated the role of parent–teenager communication and established that positive parent–teenager communication predicts lower teenage depressive symptoms. Positive communication is characterized by connectedness, familiarity, trust, flexibility, respect, family harmony, and providing active support for children, which was linked to decreased levels of internalizing difficulties [16]. In contrast, negative parent–teenager communication, such as dismissal, criticism, concealment, and mistrust, predicts the development of depressive conditions [16]. Garcia et al. [17] established that the parenting behaviors observed among parents with social anxiety, such as minimal warmth, over-involvement, and intrusive behaviors, can have a direct effect on the development of social anxiety in children. The same study also noted the significance of communication for modeling specific behavioral patterns including anxiety responses.

Nonverbal communication is also a pathway through which anxiety can be transmitted from parents to children. Some studies that have explored the impact of nonverbal communication on the development of childhood anxiety have focused on authoritative and authoritarian parenting. One factor that makes nonverbal communication salient is that it accompanies face-to-face conversations. Nonetheless, whereas verbal communication tends to be clearer, nonverbal communication presents opportunities for misinterpretation. Yet, certain gestures and expressions of positive affect and negative affect are typically less amenable to misinterpretation. Grebelsky-Litchman and Shenker [18] found that both authoritarian and authoritative parenting include the expression of nonverbal cues; the emotions conveyed by facial and bodily expressions affect the parent–child interaction, which can, in turn, affect children’s perceptions. Mullins and Duke [19] established that social anxiety is positively linked with errors and response times in threatening conditions but not in nonthreatening conditions. The same study also found that anxiety levels positively correlated with response times when an individual recognizes facial and bodily expressions of sadness, fear, and anger. Thus, although nonverbal communication is an important feature of parent–child interactions, consistent patterns of anxious behavior by parents may contribute to the development of anxiety in children [18].

### 1.2. Theoretical Framework

Social learning theory guided the current study [20]. This theory proposes that children are influenced by what they learn from their parents through communication and observation [16]. Social learning theory posits that “norms, attitudes, expectations, and beliefs arise from an interaction with the cultural environment around an individual” (p. 2, [20]). Given that parents are among the primary socialization agents, children observe and often model parental behaviors. In addition, because parents spend more time than many others with their children, consistent patterns of anxiety behavior displayed to children may contribute to the development of childhood anxiety. In sum, anxiety behavior may be adopted by children via modeling, explicit learning, and interaction with parents.

## 2. Materials and Methods

### 2.1. Participants and Procedures

A survey was administered to 121 adults (69 women and 52 men) in Saudi Arabia’s regions of Mecca, Al-Bahah, Riyadh, Medina, Tabuk, and Asir. The variables of interest were measured with items developed for this study, as presented below. Due to institutional review board restrictions, we were unable to collect detailed demographic information on the participants, including age, education, and gender. The goal of the study was communicated to the participants before they participated, and participation was voluntary.

### 2.2. Materials

Anxiety normalcy. Perceived anxiety normalcy was included as an independent variable to determine whether it is associated with childhood anxiety. A variety of anxiety disorders can be attributed to both internal and external factors [10]. The questionnaire contained 4 items used to assess perceptions of anxiety normalcy (e.g., “anxiety is a normal occurrence” and “there are varying degrees of anxiety”). As with other measures described in this section, participants indicated the strength of their belief/knowledge/understanding/experience using a 5-point scale ranging from 1 (strongly disagree) to 5 (strongly agree) for each item.

Parental anxiety. Perceptions of parental anxiety served as an independent variable. Participants answered 5 items addressing perceptions of parental anxiety, including whether parents may cause their children to develop anxiety because of how they communicate with them. Responses for each item were coded on a 5-point scale similar to the scale described in the previous paragraph.

Authoritative behavior. Perceptions of authoritative behavior were assessed as an independent variable. There was a need to determine whether flexible, facilitative, warm, firm, and supportive parents influence anxiety in children through their communication (verbal and nonverbal). This variable is linked to a parent’s personality traits and, therefore, may be expressed in parenting style. Participants were presented with a list of 4 questions addressing parenting style (e.g., “authoritative (flexible, responsive, and reciprocal) parents are not likely to cause anxiety in their children”).

Authoritarian behavior. Similarly, perceptions of authoritarian behavior were assessed as an independent variable. Certain aspects of this variable were assumed to be present in parents with anxiety, including shouting at children, being overly critical, and discouraging children’s action through fear-related information. Participants were presented with 8 items and responded to each on a 5-point scale ranging from 1 to 5, as described in the previous paragraphs. An example of an item in this measure is, “I discourage my child from doing certain things through a look or facial expression”.

Childhood anxiety. Perceptions of childhood anxiety were assessed as a key dependent variable. The critical question for this variable was whether childhood anxiety normally occurs or is learned from the environment. In the latter case, social learning theory might explain how a child learns behaviors from others (i.e., parent modeling). Participants responded to 2 items: “Children observe and model the attitude, behaviors and emotional responses of their caregivers”, and “Children are likely to become anxious if their parents are generally anxious”, with responses recorded as for previous measures described above.

## 3. Results

### 3.1. Regression Analysis

For predictive analysis purposes, the ordinal classification method was used to describe the data and investigate the relationship between a dependent variable (childhood anxiety (CA)) and different independent variables (anxiety normalcy (AN), parental anxiety (PA), authoritative behavior (AB1), and authoritarian behavior (AB2)), as shown in Table 1. The estimated values of AN (0.036), PA (2.547), AB1 (0.008), and AB2 (0.432) had significance values of 0.935, <0.001, 0.981, and 0.206, respectively. PA was strongly predictive of CA, as reflected in the large positive estimated value of 2.547. The *p*-values associated with the estimated values for AN (0.935) and AB1 (0.981) were not statistically significant. The estimate for AB2 (with *p*-value 0.205) was marginally statistically significant and had a small positive (nominal) relationship with CA as reflected in the estimated value of 0.432.

Therefore, as shown in Table 1, PA and AB2 had positive regression coefficients of 2.547 and 0.432, respectively. This means that for every one-unit increase in PA and AB2, there was a predicted increase in the log odds of a higher level on CA (significantly so for PA but only nominally for AB2). In contrast, AN and AB1 had smaller regression coefficients of 0.036 and 0.008, respectively, indicating weak or no relationships.

### 3.2. Correlation Analysis

Because the data were not normally distributed, the correlation analysis focused on Spearman rank-order (*rho*) correlations. The results of the analysis are shown in Table 2. The correlation between AN and CA of 0.116 and the correlation between AB1 and CA of 0.072 indicated weak relationships. The *p*-values, however, indicated that these correlations were not statistically significant. The correlation between PA and CA of 0.525 and the correlation between AB2 and CA of 0.137 indicated strong and weak relationships, respectively (significantly so for CA, but only nominally for AB2).

## 4. Discussion

The results indicate that perceptions of parental anxiety (significantly and strongly) and authoritarian parenting behavior (nominally or nonsignificantly) were related to childhood anxiety in a Saudi Arabian sample. In both cases, the results may not be surprising given the close relationship and frequent interactions between parents and children. Moreover, it has been established that children may learn behaviors and attitudes through observation and modeling [20]. In these cases, communication (both verbal and nonverbal) can play an important role because it is an important mode of interaction. A parent suffering from anxiety and who employs an authoritarian style can serve as a behavioral model for a child [14]. For instance, such a parent may transmit anxiety through verbal and nonverbal communication when dissuading a child from doing something (e.g., “do not climb higher; you may fall”). Verbal communication accompanied by facial expressions of worry can affect a child’s perception of the activity, leading to prospective questioning of and worry about the same activity. In addition, children tend to trust their parents and believe what their parents tell them. Furthermore, children are not fully cognitively developed to process information based on experience and make a decision appropriate to the situation. Typically, therefore, children often model verbal and nonverbal communications transmitted by their parents. This suggests that parents that display anxiety and authoritarian behavior may facilitate the development of anxiety in children.

Although this study focused on the relationship between perceptions of childhood anxiety and different variables, with parental anxiety and authoritarian behavior investigated as influences, the current findings are corroborated by a related case-control study. In a three-year case-control study, Hofman et al. [12] found that children subjected to fear-related verbal communication developed anxiety in controlled conditions, whereas positive verbal communication (which can be linked to authoritative parenting) had the opposite effect. One of the results of the current study (in both the regression and correlation analyses) was a weak (nominal) relationship between authoritative parenting and childhood anxiety. An analysis of an authoritative parenting style suggested why this form of parenting could be (weakly) related to childhood anxiety. For instance, such parents administer fair and consistent punishment (i.e., not erratic, like authoritarian parents), allow for the expression of emotions during interactions, are warm and nurturing, listen to their children, and foster autonomy and reasoning [5]. At the core of such interactions and relationships is open communication that provides a safe and enabling space for responsiveness. Authoritative parents serve as facilitators and communicate this to their children so that their children may freely express themselves. Thus, children of authoritative parents tend to have a positive disposition, appropriate social skills, confidence, emotional control, and regulation [7]. In contrast, authoritarian parents are strict, controlling, unpredictable, and inflexible. Several factors could explain this strictness and unpredictability, as these characteristics mirror anxiety symptoms such as nervousness, a sense of impending danger, panic, and irritability [9]. Whatever the parent feels, they often communicate this as fear-inducing or warning-relevant verbal and nonverbal communication. Such information is often accompanied by not only strong words but also clear facial and bodily expressions of emotions such as disappointment, disgust, and anger.

Perceived anxiety normalcy was found to have a weak (nominal) correlation with childhood anxiety. This suggests that in the current Saudi Arabian sample, anxiety was not perceived to naturally occur despite being a heterogeneous condition with related conditions such as panic disorder and social anxiety disorder. In the case of parents and children, the type of communication that occurs for each of the two parenting styles might explain, in part, why one child develops anxiety and another does not. Furthermore, in addition to these and other environmental factors (i.e., parenting style and communication), children of parents with anxiety may inherit a predisposition to develop anxiety. Thus, children with anxious parents are both genetically and environmentally predisposed to experience anxiety.

The idea that parents with anxiety can transmit this condition to children is explained by the social learning theory originating from Bandura [20]. This framework posits that children learn through observation, modeling, and imitation of other people’s behaviors and emotional reactions. This is especially expected in the case of a parent–child relationship, which tends to be closer than other relationships, irrespective of the quality of the relationship (i.e., positive or negative). Because children are still developing, they will normally identify the cues and behaviors they see in others (especially parents) and perceive these as normal, typical, or expected. For instance, if a parent is often shouting, a child who grows up in that environment will view shouting as a normal occurrence. Although social learning is a reality of life, it is influenced by the type of environment and, in this case, the type of parent with respect to the parent’s disposition and style of communication. Some parents suffer from anxiety and other conditions that could affect their children. Based on Bandura’s framework, children follow five stages in learning from others’ behaviors, namely observation, attention, retention, reproduction, and motivation [20]. Because children spend more time with their parents than with other adults, they may develop anxiety if they are subjected to consistent behavior and communication that showcases anxiety.

### Limitations, Future Directions, and Conclusions

In summary, the current findings indicate a link between the perceptions of parental anxiety and communication (verbal and nonverbal) and the development of childhood anxiety. Although similar studies have focused on verbal and nonverbal communication in Western samples, this study extended the scope to consider parent–child relationships in a Middle Eastern, Saudi Arabian, sample. Parents employ specific parenting styles depending on their personality traits. This study focused on two extreme ends of parenting style, although there are other types of parenting styles [1,2,3,4,5,6]. The parenting styles selected for this study are, in some sense, opposites of each other, and one of the parenting styles (authoritarian) shares some characteristics of a parent with anxiety [1,2,3,4,5,6]. Therefore, we investigated whether perceptions of these parenting styles were associated with childhood anxiety.

The results reported here should be considered in light of several limitations. Foremost, the study was undertaken in Saudi Arabia, and, hence, the results may not be generalizable outside Saudi Arabia. To confirm the findings of this research, prospective studies should be conducted in other countries, including other Middle Eastern countries. This is important considering that different countries and regions of the world express distinct cultures based on religious beliefs, parenting styles, etc. Second, future research might investigate the function of communication for parenting styles not addressed in the current study, such as permissive and neglectful parenting. Neglectful and permissive styles contain elements that may have contributed to better understanding parenting behavior in the current study [1,2,3,4,5,6]. Third, more longitudinal data on this topic are needed to strengthen and generalize the results because such data would enable the identification of the causal links among the variables. Fourth, confounding variables such as age and gender were not addressed in the study even though they may affect parenting styles, communication, and the development of childhood anxiety. Fifth, the current study relied on self-reported perceptions of individual experiences, knowledge, and understanding. We recognize that there may be discrepancies between parents and children in their perceptions of the variables assessed. This suggests that future research might profitably include assessments of parents and their children, allowing for dyadic analyses of linked responses. Sixth, we note that the current study was not able to address the degree to which childhood anxiety might vary with the child’s neurological status, but we recognize this is an interesting and important direction for future research, particularly in reference to the role of parental communication in affecting childhood anxiety (e.g., [21,22]). Finally, we note that future research might investigate whether and how childhood anxiety or other problems can affect the expression of parental personality and parental communication (see [22]). Disturbed personality expression might interfere with parental communication, exacerbating childhood anxiety, for example. Indeed, several studies have confirmed the presence of increased stress in parents of children with neurodevelopmental disorders compared to parents of typically developing children (e.g., [23,24]).

In spite of these limitations, this research may have implications for parents in terms of how they communicate with their children. Parents should be aware of how their communication (verbal and nonverbal) and parenting style may affect anxiety in their children. Transparent communication, active listening, warmth, and responsiveness (as characterized by authoritative parenting) is a healthier style of parenting compared with authoritarian parenting insofar as the former has a weaker correlation with the development of childhood anxiety (see also [1,2,3,4,5,6]). Parents should be careful with the words they use to communicate and their facial and bodily expression as children may model such behavior and attitudes [1,2,3,4,5,6,20]. Future studies might investigate how communication influences anxiety in different cultural contexts to determine if culture influences the development of anxiety in children, thereby extending the current research in the Saudi Arabian context to other cultural contexts.

## Figures and Tables

**Table 1:** Results of ordinal regression analysis.

						95% Conf. Interval	
	Estimate	SE	Wald	df	P	Lower bound	Upper bound
Threshold [CA = 2.00]	7.302	2.900	6.339	1	0.012	1.618	12.986
[CA = 2.50]	8.044	2.826	8.102	1	0.004	2.505	13.583
[CA = 3.00]	8.478	2.805	9.135	1	0.003	2.980	13.976
[CA = 3.50]	10.002	2.797	12.790	1	<0.001	4.520	15.483
[CA = 4.00]	12.388	2.861	18.745	1	<0.001	6.780	17.996
[CA = 4.50]	13.767	2.912	22.357	1	<0.001	8.061	19.474
AN	0.036	0.441	0.007	1	0.935	−0.829	0.901
PA	2.547	0.438	33.742	1	<0.001	1.687	3.406
AB1	0.008	0.342	0.001	1	0.981	−0.662	0.679
AB2	0.432	0.342	1.596	1	0.206	−0.238	1.102

*n* = 121; *SE* = Standard error; see text for variable definitions and abbreviations.

**Table 2:** Results of the correlation analysis.

	CA	AN	PA	AB1	AB2
CA	1.000	0.116	0.525 **	0.072	0.137
Sig. (2-tailed)	−	0.205	<0.001	0.432	0.133
AN		1.000	0.166	0.123	0.110
Sig. (2-tailed)		.	0.069	0.180	0.230
PA			1.000	0.197 *	0.095
Sig. (2-tailed)			.	0.031	0.302
AB1				1.000	−0.090
Sig. (2-tailed)				.	0.327
AB2					1.000
Sig. (2-tailed)					.

** Correlation is significant at the 0.01 level (2-tailed); * correlation is significant at the 0.05 level (2-tailed); *n* = 121; see text for variable definitions and abbreviations.

## Data Availability

Due to the nature of this research, participants did not agree to having their identified data be publicly shared.
